# Neonatal Outcomes Following a Preconception Lifestyle Intervention in People at Risk of Gestational Diabetes: Secondary Findings from the BEFORE THE BEGINNING Randomized Controlled Trial

**DOI:** 10.3390/nu17213492

**Published:** 2025-11-06

**Authors:** Md Abu Jafar Sujan, Hanna Skarstad, Guro Rosvold, Stine Lyngvi Fougner, Turid Follestad, Siri Ann Nyrnes, Kjell Salvesen, Trine Moholdt

**Affiliations:** 1 Department of Circulation and Medical Imaging, Norwegian University of Science and Technology, 7491 Trondheim, Norway; md.a.j.sujan@ntnu.no (M.A.J.S.); hanna.s.skarstad@ntnu.no (H.S.); guro.rosvold@ntnu.no (G.R.); siri.a.nyrnes@ntnu.no (S.A.N.); 2 Department of Women’s Health, St. Olav’s Hospital, 7030 Trondheim, Norway; pepe.salvesen@ntnu.no; 3 Department of Clinical and Molecular Medicine, Norwegian University of Science and Technology, 7491 Trondheim, Norway; stine.fougner@ntnu.no; 4 Department of Endocrinology, St. Olav’s Hospital, 7030 Trondheim, Norway; 5 Department of Public Health and Nursing, Norwegian University of Science and Technology, 7491 Trondheim, Norway; turid.follestad@ntnu.no; 6 Children’s Clinic, St. Olav’s Hospital, 7030 Trondheim, Norway

**Keywords:** preconception lifestyle intervention, time-restricted eating, exercise training, neonatal health, birth weight

## Abstract

**Objectives:** Gestational diabetes mellitus (GDM), particularly when combined with overweight or obesity, is associated with adverse neonatal outcomes such as high birth weight and increased adiposity. We determined the effect of a preconception lifestyle intervention initiated before and continued throughout pregnancy on neonatal, birth-related, and body composition outcomes at birth and 6–8 weeks of age in children of participants in the BEFORE THE BEGINNING randomized controlled trial. **Methods:** People (*N* = 167) at increased risk of GDM and planning pregnancy were randomly allocated 1:1 to intervention or control. The intervention included time-restricted eating and exercise training. Time-restricted eating involved consuming all energy within ≤10 h/day, ≥5 days per week, and the amount of exercise was set using a heart rate-based physical activity metric (Personal Activity Intelligence, PAI), with the goal of ≥100 weekly PAI points. The main outcome of interest in this report was the proportion of infants with birth weight > 4.0 kg. **Results:** Among 106 live births, 21% (11/53) of infants in the intervention group and 28% (15/53) in the control group had birth weight > 4 kg (*p* = 0.367). Mean birth weight did not differ significantly between groups (mean difference −159.3 g, 95% confidence interval −375.7 to 57.2, *p* = 0.148). No significant between-group differences were found for additional neonatal, birth-related, or early postnatal body composition outcomes. **Conclusions:** In this secondary analysis, we found no evidence of effects of a preconception lifestyle intervention on the risk of macrosomia or neonatal body composition.

## 1. Introduction

The global prevalence of overweight and obesity has increased markedly over the past few decades. Currently, an estimated 44% of all adult women are classified as overweight or obese [1], conditions that substantially elevate the risk of pregnancy complication, including gestational diabetes mellitus (GDM) [2]. GDM, particularly when combined with elevated maternal body mass index (BMI), is associated with adverse perinatal outcomes, such as fetal overgrowth, neonatal obesity, and a range of obstetric complications, including preterm birth, shoulder dystocia, neonatal asphyxia, induction of labor, caesarean or instrumental delivery, and postpartum hemorrhage [3,4,5,6]. Moreover, offspring of affected pregnancies are predisposed to long-term health consequences, including childhood and adolescent obesity and type-2 diabetes later in life [7,8].

While exercise only interventions have been associated with a 39% reduction in odds of having babies with birth weight > 4 kg [9], several systematic reviews and meta-analyses have concluded that lifestyle interventions in pregnancy exert minimal or no effects on birth weight or other neonatal outcomes [9,10,11,12,13,14,15]. Notably, most lifestyle interventions to prevent GDM and the associated adverse offspring outcomes have been initiated at gestational weeks 16–20, thereby missing a critical window to optimize maternal health prior to conception and during embryonic and early fetal development [16]. Observational evidence suggests that a healthy maternal lifestyle before pregnancy is associated with favorable embryonic development and confer long-term health benefits for the offspring [16]. Nevertheless, only a limited number of randomized controlled trials have investigated the effects of preconception lifestyle interventions on neonatal outcomes [17,18,19,20]. Among these, one trial demonstrated a significant reduction in the proportion of neonates born large for gestational age (LGA) in the intervention group [17], whereas others reported no effect [18,19,20].

Emerging evidence suggests that alternative lifestyle intervention strategies, such as time-restricted eating and high-intensity exercise training, can improve cardiometabolic health in people with cardiometabolic disorders [21,22,23,24,25]. Although both strategies are feasible during pregnancy [26,27,28], their effects on maternal and neonatal health outcomes remain unexplored.

In the BEFORE THE BEGINNING trial [29], we included participants who were planning pregnancy and identified as being at increased risk of GDM. The intervention consisted of time-restricted eating and exercise training, initiated before conception and continued throughout pregnancy. The primary findings demonstrated no significant effect on maternal glucose tolerance but a reduction in weight and fat-mass again by gestational week 28 [30]. Here we report secondary neonatal and birth-related outcomes from the trial. We hypothesized that the intervention would reduce the proportion of neonates with a birth weight > 4 kg and would favorably influence additional birth-related outcomes.

## 2. Materials and Methods

### 2.1. Study Design and Setting

**Figure 1:**
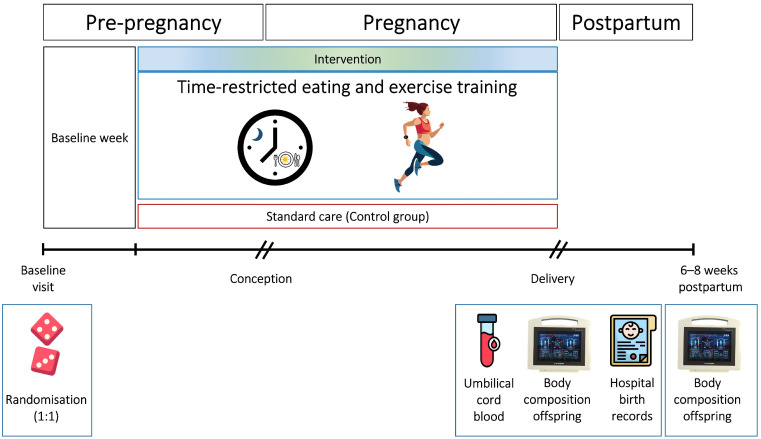
Study design. After baseline assessments, the participants were randomly allocated (1:1) to a lifestyle intervention or a standard care control group. The intervention consisted of time-restricted eating and exercise training, started before, and continued throughout pregnancy. We obtained birth weight and secondary neonatal and birth-related outcomes from hospital records and collected umbilical cord blood samples immediately after delivery. We assessed infant body composition within 72 h after birth and at 6–8 weeks of age.

The BEFORE THE BEGINNING was a single-center randomized controlled trial conducted at the Norwegian University of Science and Technology (NTNU), in collaboration with St. Olav’s Hospital, both located in Trondheim, Norway. We registered the trial in ClinicalTrials.gov (NCT04585581) on 25 September 2020. All participants signed an informed written consent (Supplementary File S1). A detailed study protocol including protocol modifications after trial commencement [29] and the main findings [30] of the trial have been published previously. Figure 1 provides an overview of the study design.

### 2.2. Recruitment and Participants

We recruited participants through advertisements on social media, hospital and university websites, local stores, and public spaces. In November 2022, we expanded recruitment by using population data from the Norwegian Tax Administration to distribute electronic invitations. Eligible participants were females aged 18–39 years who planned to conceive within 6 months, understood oral and written Norwegian or English, and met the Norwegian guidelines’ criteria for elevated GDM risk [31]. Our exclusion criteria were: ongoing pregnancy, trying to conceive ≥ 6 cycles, known diabetes (type 1 or 2), shift work involving night shifts > 2 days/week, a history of hyperemesis, known cardiovascular diseases, high-intensity exercise > 2 times/week in the last 3 months, habitual eating window ≤ 12 h/day, previous bariatric surgery, or any other reason which according to the researchers made the potential participant ineligible.

### 2.3. Randomization and Blinding

After baseline assessments, we randomly allocated the participants (1:1) to the intervention or a standard care control group, stratified by GDM in a previous pregnancy (yes/no). Detailed information about methods used for random sequence generation, concealment, and block sizes were published previously [29,30]. The participants, the investigators, or the infant body composition outcome assessors were not blinded to randomization. For the neonatal and birth-related outcomes, including the main outcome of interest, outcome assessors were blinded.

### 2.4. Intervention and Adherence

Participants in the intervention group underwent a lifestyle program combining time-restricted eating and exercise training that was initiated preconception and continued throughout pregnancy. Time-restricted eating involved limiting the daily energy intake to ≤10 h, ending no later than 19:00, on ≥5 days per week. We did not describe diet composition or energy intake, and the participants were permitted to consume non-caloric beverages outside the eating window. Every 8th week, participants recorded their eating window in a study handbook. Exercise training was guided by Personal Activity Intelligence (PAI), a heart rate-based measure of physical activity [32]. We instructed the participants to achieve and maintain ≥100 weekly PAI points through high-intensity endurance exercise, modified as appropriate for pregnancy. Details about the exercise intervention can be found in the published study protocol [29] and in Supplementary File S2. Training was primarily unsupervised, supplemented by supervised sessions at 2 and 8 weeks after baseline and on request. We monitored adherence to exercise training using smartwatches (Amazfit GTS, Huami, Hefei, China/Polar Ignite 2, Polar Electro Oy, Kempele, Finland), which transmitted activity data to the research team. Control group participants received standard care and were asked to maintain their habitual physical activity and diet.

### 2.5. Experimental Procedures and Outcome Measures

In this report, we present neonatal and birth-related outcomes and body composition of infants within 72 h of birth and at 6–8 weeks of age. The main outcome of interest in this report was frequency of birth weight > 4 kg. From hospital records, we obtained the following birth-related outcomes: mode of delivery, perineal tear, episiotomy, shoulder dystocia, postpartum hemorrhage, and duration of hospital stay. Neonatal outcomes included birth weight, length, head circumference, APGAR score, and gestational age at birth. The APGAR score is a standardized tool used to assess neonatal health immediately after birth, comprising five criteria (skin color, heart rate, reflexes, muscle tone, and respiration), each scored from 0 to 2, with a maximum total score of 10. A score of 7–10 at 5 min after birth is considered normal [33]. We therefore report the frequency of APGAR score < 7 at 5 min. We calculated the frequency of preterm birth, defined as delivery before 37 gestational weeks, from gestational age at birth.

Midwives at St. Olav’s Hospital collected umbilical cord blood immediately after birth, prior to delivery of the placenta. After resting in room temperature for 30 min, the blood samples were centrifuged at 3100 RPM at 18 °C for 10 min. Serum was aliquoted and stored in −80 °C until analysis. Serum insulin C-peptide concentrations were measured by electrochemiluminescence immunoassay using a Roche Cobas Pro e801 analyzer (Roche Diagnostics, Basel, Switzerland), and glucose concentrations were measured by photometric assay using a Siemens Atellica CH930 analyzer (Siemens Healthineers, Erlangen, Germany), both performed at the St. Olavs’s Hospital laboratories.

We estimated infant body composition using bioelectrical impedance analysis (BIA, BioScan touch i8-nano, Maltron, Essex, UK) within 72 h of birth and again at 6–8 weeks of age. Prior to the measurement, we entered gestational age at birth, age at measurement, sex, ethnicity, length, and body weight into the Bioscan device. With the infants in a supine position, we attached two surface electrodes on the dorsal surface of the right hand (wrist and metacarpal level) and two on the right foot (ankle and metatarsal level). The BioScan device passes a low-amplitude, multi-frequency electrical current through the body, and calculates resistance and reactance values to estimate total body water, fat-free mass, fat mass and muscle mass using age- and sex-specific equations. Trained study personnel performed all measurements to ensure accuracy and repeatability. The procedure was non-invasive, brief, and well tolerated by the infants.

### 2.6. Statistical Analysis

We calculated the sample size for the BEFORE THE BEGINNING trial based on the primary outcome measure: 2 h plasma glucose concentration during a 75 g oral glucose tolerance test (OGTT) in gestational week 28. To detect a between-group difference of 1.0 mmol/L, with a standard deviation (SD) of 1.3, 90% power, and a two-sided significance level of 0.05, we needed 74 participants. To account for non-conception (~50%), drop-outs (10–20%), and to increase statistical power for secondary analysis, we initially aimed to include 260 participants. We stopped the recruitment after 167 participants, which was considered sufficient to compensate for attrition and non-conception, as described previously [29]. No separate sample size calculation was done for the secondary outcomes reported in this paper.

We performed intention-to-treat analyses including the 106 participants who gave birth. For between-group comparisons of outcome measures at birth, we used two-tailed independent samples *t*-tests and Mann–Whitney U-tests for continuous data, and χ^2^ or Fisher’s exact tests for categorical data. We assessed data normality using the Shapiro–Wilk test and visual inspection of normal QQ plots. Estimated effects are reported as mean differences or risk ratios in the intervention group compared with the control group, with corresponding 95% confidence intervals (CIs) and *p*-values. For rare categorical outcomes, we present descriptive statistics without formal testing. We evaluated changes in infant body composition from birth to 6–8 weeks of age using linear mixed models with time, group and group x time interaction as fixed effects and participant ID as a random effect.

Since this paper reports multiple secondary outcomes, we pragmatically applied a significance threshold of 0.01, to reduce the risk of false positives from multiple comparisons. Although formals methods (e.g., Bonferroni correction) for *p*-value adjustment are commonly used to account for multiple comparisons, they can be overly conservative or complex in studies with correlated outcomes [34], and were therefore not applied in our analyses. We also performed per-protocol analyses according to a prespecified statistical analysis plan [29]. The statistical analyses were performed using IBM SPSS Version 29.0.1.0 (IBM Corp., Armonk, NY, USA).

### 2.7. Patient and Public Involvement

We involved users (reproductive-aged females with overweight/obesity) in the planning and implementation of the study. Participant representatives were not involved in the recruitment process. We organized multiple interactive digital meetings and workshops to discuss barriers to participation, motivation, engagement, data collection methods, recruitment strategies, and adherence throughout the study.

## 3. Results

**Figure 2:**
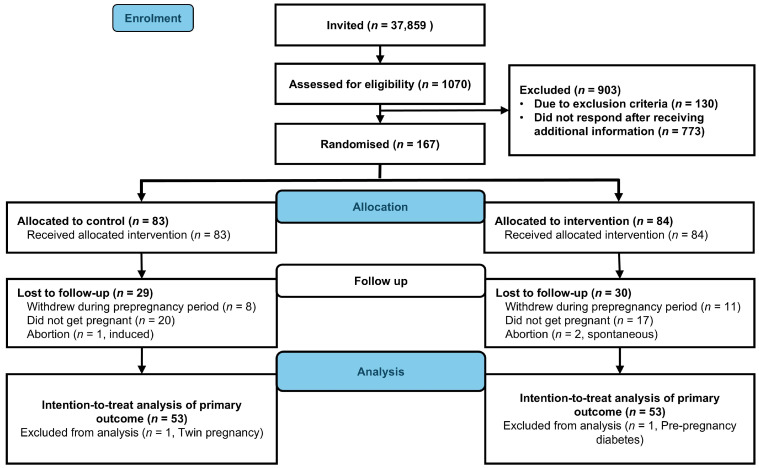
Flowchart of participants (CONSORT Flow diagram).

We enrolled 167 participants between 2 October 2020 and 12 May 2023. Within the specified time frame, 111 participants became pregnant (control, *n* = 55, intervention, *n* = 56, Figure 1). We excluded data from one participant in the intervention group due to prepregnancy diabetes and one control participant due to twin pregnancy. Neonatal and birth-related outcomes were missing for three participants. Two participants in the intervention group experienced spontaneous abortion, and one in the control group underwent induced abortion. In total, we included data from 106 live births (control, *n* = 53, intervention, *n* = 53) in the intention-to-treat analyses of neonatal and birth-related outcomes (Figure 2). The baseline characteristics were comparable between groups (Table 1).

### 3.1. Neonatal Outcomes

**Figure 3:**
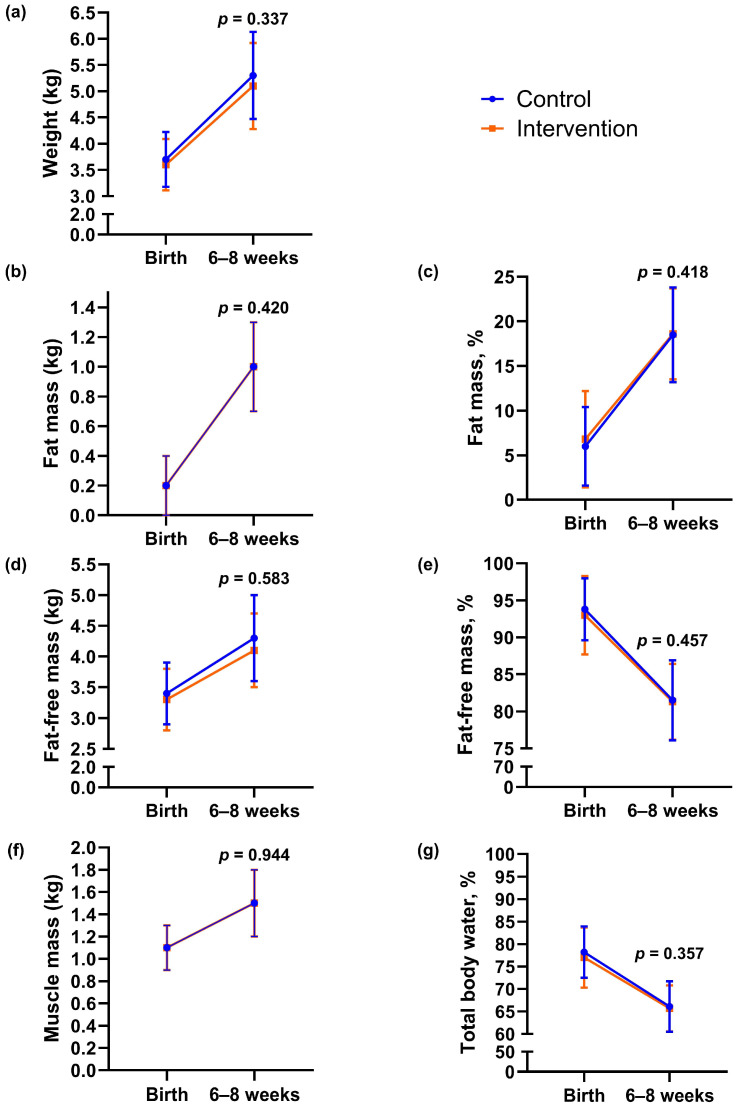
Body composition outcomes of infants at birth and 6–8 weeks of age according to group. (**a**) Body weight, (**b**) fat mass, (**c**) percentage of fat, (**d**) fat-free mass, (**e**) percentage of fat-free mass, (**f**) muscle mass, (**g**) hydration (total body water percentage). Data are presented as observed means, and error bars show mean ± 1 standard deviation (SD). The lines represent changes in each group from birth to 6–8 weeks of age. The *p*-values were calculated for between-group differences using linear mixed model.

In the intervention group, 21% (11/53) of newborns had birth weight > 4 kg, compared with 28% (15/53) in the control group (*p* = 0.367, Table 2). Birth weight did not differ significantly between groups (mean difference −159.3 g, 95% CI −375.7 to 57.2, *p* = 0.148). We found no statistically significant between-group differences in other neonatal outcomes or in body composition outcomes assessed within 72 h of delivery (Table 2). One newborn in the control group had an APGAR score < 7 at 5 min after delivery, whereas none in the intervention group did (Table 2). We estimated infant body composition at a mean of 33 h (SD 1.1) after delivery and again at 7 weeks (SD 1.4). From birth to 6–8 weeks, there were no statistically significant between-group differences in changes in infant body weight, fat mass, fat-free mass, percentage of fat mass and fat-free mass, muscle mass, or hydration (Figure 3 and Appendix A Table A1).

### 3.2. Birth-Related Outcomes

There were no statistically significant differences between groups in mode of delivery, perineal tear, episiotomy, postpartum hemorrhage, or length of the hospital stay (Table 3). One case of shoulder dystocia occurred in the control group, and none in the intervention group (Table 3). Nine of 53 participants (17%) in the intervention group and four of 53 (8%) in the control group were delivered by caesarean section (relative risk 2.3, 95% CI 0.7 to 6.9, *p* = 0.139). The median length of hospital stay was 3 days in both groups.

### 3.3. Per Protocol Analysis

For the per-protocol analysis, we included intervention participants who obtained ≥ 75 weekly PAI-points, reported adherence to ≤10 h time-restricted eating on at least two of four registered days in each registration during the preconception period, and delivered a singleton live baby. Twenty-three of 53 (43%) participants in the intervention group met these criteria. In total, we included 75 participants (control, *n* = 52, intervention, *n* = 23) in the per-protocol analyses. The per-protocol results were consistent with those from the intention-to-treat analysis (Appendix A Table A2, Table A3 and Table A4).

## 4. Discussion

### 4.1. Main Findings and Comparison with Previous Studies

In the BEFORE THE BEGINNING trial, a preconception intervention combining time-restricted eating and exercise training did not significantly affect birth weight, infant body composition, or other birth related outcomes. Our findings align with previous preconception lifestyle intervention studies in populations at high risk of GDM, which similarly reported no significant impact on neonatal outcomes [18,19,20]. We observed a small, non-significant reduction in mean birth weight (159 g) and proportion of macrosomia (7% absolute risk reduction) in the intervention group compared with the control group, consistent with findings from two prior studies of combined preconception dietary and exercise interventions [19,20]. While not statistically significant, these reductions may nonetheless be clinically meaningful. Considering the limited power for secondary outcomes, larger studies are needed to find evidence of a significant effect on birth weight and macrosomia. Maternal prepregnancy BMI and weight gain during pregnancy influence birth weight [35]. In our study, more than 80% of the participants were enrolled due to elevated BMI (≥25 kg/m^2^). Although weight loss before or during pregnancy was not specifically targeted, participants in the intervention group gained significantly less body weight and fat mass in late pregnancy [30]. These changes may not have been sufficient to improve maternal glycemic control or other cardiometabolic outcomes influencing neonatal outcomes.

Unlike our trial, previous preconception trials have emphasized weight loss prior to pregnancy and gestational weight maintenance [17,18,19,20]. Notably, a 12-week very-low-energy diet intervention reduced the prevalence of LGA infants from 24% in the control group to 4% in the intervention group among participants with a BMI between 30 and 55 kg/m^2^. This intervention, consisting of a preconception very-low-energy diet (800 kcal/day) for 12 weeks followed by an energy-balanced diet combined with physical activity (>10,000 steps/day), induced significant weight loss before conception [17]. The resulting improvement in maternal metabolic status may have optimized the intrauterine environment, thereby decreasing the risk of LGA births in the intervention group [17].

Lifestyle interventions initiated during pregnancy have produced inconsistent effects on birth weight outcomes. A meta-analysis showed that exercise-only interventions were more effective than combined exercise and co-interventions, reducing the odds of macrosomia (birth weight > 4 kg) by 39% [9]. Most studies included in that meta-analysis relied on unsupervised exercise or counselling-based approaches, which were associated with lower adherence. Similarly, the intervention in our trial was mainly unsupervised. During preconception, nearly half of the study participants adhered to the ≤10 h eating window (49%) and 43% achieved ≥100 weekly PAI points. However, adherence to both time-restricted eating and exercise declined as pregnancy progressed, dropping to 38% and 15%, respectively, by the third trimester [30]. Declining adherence, as well as a predominantly highly educated, white participant group, and reduced statistical power in the secondary analyses may have obscured the true effects of the intervention and limited the generalizability of our findings.

Our study, consistent with two previous preconception trials and several pregnancy lifestyle intervention studies, did not demonstrate statistically significant effects of the intervention on adverse neonatal or birth-related outcomes [9,10,18,20,36,37,38]. In contrast, the very-low-energy diet intervention study reported a significantly lower risk of a composite of obesity-related adverse pregnancy outcomes in the intervention group compared with the control group [17]. The overall incidence of adverse neonatal and birth-related outcomes in our study was low, limiting the statistical power to detect meaningful between group differences. Complications such as shoulder dystocia, low APGAR scores, preterm birth, episiotomy, caesarean section, and postpartum hemorrhage occurred infrequently in both groups. Notably, caesarean section rates in our study were lower than those reported in previous pre-pregnancy studies involving participants with overweight or obesity or those at increased risk of GDM [17,18,20]. Whereas comparable populations have shown rates ranging from 20% to 52%, our study observed rates of 17% (9/53) in the intervention group and 8% (4/53) in the control group. This lower-than-expected incidence may reflect differences in clinical practice, participant characteristics, or the relatively healthy baseline status of our cohort despite elevated GDM risk.

Cord blood glucose and insulin C-peptide concentrations reflect maternal metabolic status and glycemic and insulin homeostasis during pregnancy [39]. Consistent with the findings from the LIMIT [40] and UPBEAT trials [41], we found no statistically significant differences in cord blood glucose or insulin C-peptide concentrations between newborns of participants in the intervention and control groups.

There were no significant between-group differences in infant body composition outcomes measured within 72 h of birth, consistent with the conclusions of two meta-analyses of lifestyle intervention studies during pregnancy [9,35]. Of note, the estimated neonatal mean fat percentage in our cohort was lower (6.4%) than that reported in several other studies of lifestyle interventions in women with overweight/obesity (~10–14%) [42,43,44,45,46], which could potentially be attributed to the use of BIA. BIA tends to underestimate fat mass and overestimate fat-free mass compared with gold-standard methods such as air-displacement plethysmography and dual-energy X-ray absorptiometry in pediatric populations [47]. Although BIA has limitations in accuracy at the individual level, it was selected for practical reasons: the method is non-invasive, cost-effective, rapid, feasible in clinical studies, and suitable for repeated measurements in infants [48,49]. Importantly, the same standardized protocol and equipment were used for all participants, minimizing systematic bias between groups. While some random measurement error cannot be excluded, such errors would likely have attenuated rather than exaggerated true between-group differences.

Infant body composition outcomes did not change significantly between groups at 6–8 weeks of age. Our pooled data indicated a three-fold increase in percentage of body fat from birth, accounting for ~47% of total weight gain. Early infant feeding practices influence growth trajectory and body composition, and exclusively breastfed infants experience a greater increase in percentage of body fat compared with formula-fed infants [50,51,52,53]. In our study, breastfeeding practices were similar between the groups, and a high proportion of infants (35/42, 83%) were exclusively breastfed, which may have contributed to the observed increase in fat percentage. In contrast, in an Australian cohort of infants born to mothers with a healthy BMI and no GDM, fat percentage doubled after 6 weeks, contributing to 40% of total weight gain [50]. They observed no difference between breastfed and other infants in fat mass increase as a proportion of weight gain at 6 weeks and 3 months, but exclusively breastfed infants had a greater increase at 4.5 months. Body composition changes rapidly during infancy, and accelerated fat mass gain and rapid growth during this period is associated with increased risk of obesity later in childhood [54], suggesting that early growth patterns influence long-term health outcomes. Follow-up of these children may provide valuable insight into the impact of early body composition changes on future cardiometabolic health.

### 4.2. Limitations

A major limitation of the study was declining adherence during pregnancy despite good adherence during the preconception period [30]. The primarily unsupervised nature of the intervention was intentionally designed to reflect real-world implementation. However, limited monitoring may have impacted adherence and overall effectiveness of the intervention. We acknowledge that the risk of performance bias cannot be ruled out because of the non-blinding of participants. However, the neonatal and birth-related outcomes assessors were blinded to randomization. We obtained these data from standardized medical records, which are objective in nature and reduce subjectivity in outcome assessment and detection bias. We could not blind the assessors of infant body composition outcomes due to a limited number of researchers in the trial. The analyses of these outcomes were secondary, and the incidence of some outcomes was very low. Furthermore, we calculated the sample size based on the primary outcome of the BEFORE THE BEGINNING trial, not the secondary outcomes reported here, which increases the risk of Type II errors and may have obscured true effects. We estimated infant body composition at two time points in early infancy, providing a longitudinal perspective rather than a single cross-sectional measurement. However, the use of BIA to estimate body composition represents a limitation due to its lower precision compared with gold-standard methods [55].

## 5. Conclusions

In the BEFORE THE BEGINNING trial, a preconception intervention combining time-restricted eating and exercise training had no statistically significant effect on neonatal outcomes, including birth weight, body composition at birth or 6–8 weeks of age, or other birth-related outcomes. Although the intervention led to beneficial changes in maternal body composition during pregnancy, these changes did not translate into measurable improvements in neonatal or birth-related outcomes. Given the rising global prevalence of obesity and diabetes, continued investigation into preconception lifestyle interventions remains a public health priority. Future studies should aim to include more diverse populations, stratify participants by BMI and metabolic risk, and ensure sufficient statistical power to analyze neonatal outcomes. Longitudinal follow-up beyond infancy will be critical to determine whether early lifestyle interventions confer sustained benefits for offspring health.

## Figures and Tables

**Table 1:** Baseline characteristics of participants who delivered a singleton live baby, according to group allocation.

	Control (*n* = 53)	Intervention (*n* = 53)
Age, years	29.5 (3.1)	29.8 (3.4)
Weight, kg	80.0 (12.8)	80.8 (15.9)
Body mass index, kg/m^2^	28.5 (4.4)	29.1 (4.9)
Waist circumference, cm	93.3 (11.2)	92.5 (12.9)
Fat percentage, %	37.1 (7.1)	36.3 (7.9)
Systolic blood pressure, mmHg	120 (10)	119 (8)
Diastolic blood pressure, mmHg	79 (7)	79 (6)
HbA1c, mmol/mol	34.1 (2.8)	34.3 (2.8)
Fasting glucose, mmol/L	5.0 (0.4)	5.0 (0.4)
Total cholesterol, mmol/L	4.5 (0.8)	4.6 (0.8)
HDL cholesterol, mmol/L	1.4 (0.3)	1.4 (0.3)
LDL cholesterol, mmol/L	3.0 (0.8)	3.0 (0.8)
Triglycerides, mmol/L	1.0 (0.5)	1.0 (0.6)
Parity, total number of births, *n* (%)		
0	27 (51)	27 (51)
1	20 (38)	23 (43)
2	6 (11)	3 (6)
Education level, *n* (%)		
Completed compulsory schooling and upper secondary school	5 (9)	10 (19)
Completed university education, less than 4 years	18 (34)	14 (26)
Completed university education, 4 years or more	30 (57)	29 (55)
Ethnic origin, *n* (%)		
Europe	47 (89)	47 (89)
Africa and Middle East	2 (4)	0 (0)
Asia	3 (6)	4 (8)
North America	0 (0)	1 (2)
Latin America	1 (2)	1 (2)
Reason for inclusion *, *n* (%)		
Body mass index ≥ 25 kg/m^2^	45 (85)	44 (83)
GDM in a previous pregnancy	1 (2)	1 (2)
Family history of diabetes	14 (26)	15 (28)
Previous newborn > 4.5 kg	0 (0)	1 (2)

Data are presented as means of observed values with standard deviations (SDs) or frequencies (*n*) and percentages (%) of participants. Missing: number of participants with missing data in each group is 0 to 4 for all variables. HbA1c = Glycated hemoglobin, HDL = High-density lipoprotein cholesterol, LDL = Low-density lipoprotein cholesterol, GDM = Gestational diabetes mellitus. * Some fulfilled more than one reason.

**Table 2:** Neonatal outcomes at birth.

Outcome	Control *(n* = 53)	Intervention (*n* = 53)	Estimate	95% CI	*p*
Weight, g	3702.5 (517.8)	3543.3 (602.7)	−159.3	−375.7 to 57.2	0.148 ^a^
Length, cm	50 (49, 51)	50 (49, 52)	-	-	0.930 ^b^
Head circumference, cm	36.0 (35, 37)	35 (35, 37)	-	-	0.302 ^b^
Birth weight > 4 kg, *n* (%)	15 (28)	11 (21)	0.7	0.4 to 1.4	0.367 ^c^
APGAR < 7 at 5 min, *n* (%)	1 (2)	0 (0)	-	-	-
Preterm birth, *n* (%)	1 (2)	2 (4)	-	-	-
Gestational age at birth, weeks + days	40 + 3 (39 + 2, 41 + 1)	40 + 0 (39 + 0, 41 + 0)	-	-	0.319 ^b^
Serum glucose, mmol/L *	5.6 (1.3)	5.6 (1.3)	0.0	−0.6 to 0.6	0.997 ^a^
Serum insulin C-peptide, nmol/L *	0.5 (0.5, 0.7)	0.5 (0.5, 0.6)	-	-	0.367 ^b^
Fat mass, kg **	0.2 (0.1, 0.3)	0.2 (0.1, 0.3)	-	-	0.690 ^b^
Fat mass, % **	4.8 (2.9, 7.2)	5.5 (3.6, 8.0)	-	-	0.622 ^b^
Fat-free mass, kg **	3.5 (1.5)	3.3 (1.5)	−0.1	−0.3 to 0.1	0.218 ^a^
Fat-free mass, % **	95.2 (92.9, 97.0)	94.5 (92.0, 96.4)	-	-	0.605 ^b^
Muscle mass, kg **	1.1 (0.2)	1.1 (0.2)	−0.1	−0.1 to 0.0	0.196 ^a^
Hydration (TBW), % **	79.6 (76.3, 81.8)	78.6 (75.6, 81.2)	-	-	0.449 ^b^

Data are observed mean and standard deviations (SDs) or medians and 25- and 75-percentiles (quartiles) or frequencies (*n*) and percentages (%). Results are presented as mean differences or relative risks (RRs) with corresponding 95% confidence intervals (CIs) and *p*-values. TBW = Total body water. ^a^ Independent samples *t*-test, ^b^ Mann–Whitney U-test, ^c^ Χ^2^ test. * Control, *n* = 31, Intervention, *n* = 37, ** Control, *n* = 49, Intervention, *n* = 45.

**Table 3:** Birth-related outcomes.

Outcome	Control *(n* = 53)	Intervention (*n* = 53)	Relative Risk	95% CI	*p*
Normal vaginal delivery, *n* (%)	47 (89)	42 (79)	0.9	0.8 to 1.1	0.186 ^b^
Induced labor, *n* (%)	10 (19)	11 (21)	1.1	0.5 to 2.4	0.807 ^b^
Instrumental delivery *, *n* (%)	2 (4)	2 (4)	-	-	-
Caesarean section, *n* (%)	4 (8)	9 (17)	2.3	0.7 to 6.9	0.139 ^b^
Perineal tear *, grade 3–4, *n* (%)	1 (2)	1 (2)	-	-	-
Episiotomy *, *n* (%)	5 (10)	2 (5)	0.4	0.1 to 2.2	0.440 ^a^
Shoulder dystocia, *n* (%)	1 (2)	0 (0)	-	-	-
Postpartum hemorrhage, *n* (%)	8 (15)	6 (11)	0.8	0.3 to 2.0	0.566 ^a^
Length of hospital stay, days	3 (2, 4)	3 (2, 4)	-	-	0.247 ^c^

Data are presented as frequencies (*n*) and percentages (%) or medians and quartiles. Results are presented as relative risks (RRs), with corresponding 95% confidence intervals (CIs) and *p*-values. ^a^ Fisher’s exact test, ^b^ Χ^2^ test, ^c^ Mann–Whitney U-test. * Only for vaginal deliveries (control, *n* = 48, intervention, *n* = 44).

## Data Availability

All individual deidentified participant data and statistical codes are available on Zenodo data repository: 10.5281/zenodo.17130011. For problems accessing the data, please contact the corresponding author.

## Supplementary Materials

The following supporting information can be downloaded at: https://www.mdpi.com/article/10.3390/nu17213492/s1, Supplementary File S1: Informed written consent; Supplementary File S2: Exercise options.

## Abbreviations

The following abbreviations are used in this manuscript:

GDM	Gestational diabetes mellitus
BMI	Body mass index
PAI	Personal Activity Intelligence
LGA	Large for gestational age
BIA	Bioelectrical impedance analysis
SD	Standard deviation
CI	Confidence interval
HbA1c	Glycated hemoglobin
HDL	High-density lipoprotein cholesterol
LDL	Low-density lipoprotein cholesterol

## Appendix A

### Appendix A.1

**Table A1:** Body composition outcomes of infants at birth and 6–8 weeks of age.

Outcome	Time	Control *(n* = 49)	Intervention (*n* = 45)	Estimate	95% CI	*p*
Weight, kg	At birth	3.7 (0.5)	3.6 (0.5)			
	6–8 weeks	5.3 (0.8)	5.1 (0.8)	−0.1	−0.4 to 0.1	0.337
Fat mass, kg	At birth	0.2 (0.2)	0.2 (0.2)			
	6–8 weeks	1.0 (0.3)	1.0 (0.3)	−0.1	−0.2 to 0.1	0.420
Fat mass, %	At birth	6.0 (4.4)	6.8 (5.4)			
	6–8 weeks	18.5 (5.3)	18.6 (5.1)	−1.0	−3.4 to 1.4	0.418
Fat-free mass, kg	At birth	3.4 (0.5)	3.3 (0.5)			
	6–8 weeks	4.3 (0.7)	4.1 (0.6)	−0.1	−0.3 to 0.2	0.583
Fat-free mass, %	At birth	93.8 (4.2)	93.0 (5.3)			
	6–8 weeks	81.5 (5.4)	81.3 (5.1)	0.9	−1.5 to 3.3	0.457
Muscle mass, kg	At birth	1.1 (0.2)	1.1 (0.2)			
	6–8 weeks	1.5 (0.3)	1.5 (0.3)	0.0	−0.1 to 0.1	0.944
Hydration (TBW), %	At birth	78.2 (5.7)	77.0 (6.7)			
	6–8 weeks	66.1 (5.6)	65.7 (5.1)	1.3	−1.5 to 4.0	0.357

Data are presented as observed means and standard deviations (SDs) of outcomes measured within 72 h of delivery and at 6–8 weeks of age. Results from linear mixed model analyses are presented as estimated mean differences (estimates) in intervention group compared with control group, with corresponding 95% confidence intervals (CIs) and *p*-values.

### Appendix A.2

**Table A2:** Neonatal outcomes at birth (Per-protocol analysis).

Outcome	Control *(n* = 53)	Intervention (*n* = 23)	Estimate	95% CI	*p*
Weight, g	3702.5 (517.8)	3734.3 (451.4)	31.8	−216.4 to 280.0	0.799 ^a^
Length, cm	50.1 (2.1)	50.9 (1.9)	0.7	−0.3 to 1.8	0.158 ^a^
Head circumference, cm	36 (35, 37)	36 (35, 37)	-	-	0.840 ^b^
Birth weight > 4 kg, *n* (%)	15 (28)	6 (26)	0.9	0.4 to 2.1	0.843 ^c^
APGAR < 7 at 5 min, *n* (%)	1 (2)	0 (0)	-	-	-
Preterm birth, *n* (%)	1 (2)	0 (0)	-	-	-
Gestational age at birth, weeks + days	40 + 3 (39 + 2, 41 + 1)	40 + 1 (39 + 1, 41 + 2)	-	-	0.856 ^b^
Serum glucose, mmol/L *	5.6 (1.3)	5.8 (1.4)	0.2	−0.6 to 1.0	0.580 ^a^
Serum C peptide, nmol/L *	0.5 (0.5, 0.7)	0.5 (0.5, 0.6)	-	-	0.632 ^b^
Fat mass, kg **	0.2 (0.1, 0.3)	0.2 (0.1, 0.3)	-	-	0.967 ^b^
Fat mass, % **	4.8 (2.9, 7.2)	4.8 (2.9, 9.0)	-	-	0.877 ^b^
Fat-free mass, kg **	3.4 (0.5)	3.4 (0.4)	−0.1	−0.3 to 0.2	0.589 ^a^
Fat-free mass, % **	95.2 (92.9, 97.0)	95.2 (91.0, 97.0)	-	-	0.948 ^b^
Muscle mass, kg **	1.1 (0.2)	1.2 (0.2)	−0.1	−0.1 to 0.0	0.619 ^a^
Hydration (TBW), % **	79.3 (76.2, 81.7)	79.8 (74.2, 82.1)	-	-	1.000 ^b^

Data are presented as means and standard deviations (SDs) or medians and 25- and 75-percentiles (quartiles) or frequencies (*n*) and percentages (%). Results are presented as mean differences or relative risks (RR) with corresponding 95% confidence intervals (CIs) and *p*-values. TBW = Total body water. ^a^ Independent samples *t*-test, ^b^ Mann–Whitney U-test, ^c^ Χ^2^ test. * Control, *n* = 31, Intervention, *n* = 19, ** Control, *n* = 49, Intervention, *n* = 21.

### Appendix A.3

**Table A3:** Body composition outcomes of infants at birth and 6–8 weeks of age (Per-protocol analysis).

Outcome	Time	Control *(n* = 49)	Intervention (*n* = 21)	Estimate	95% CI	*p*
Weight, kg	At birth	3.7 (0.5)	3.6 (0.4)			
	6–8 weeks	5.3 (0.8)	5.2 (0.8)	−0.1	−0.4 to 0.2	0.360
Fat mass, kg	At birth	0.2 (0.2)	0.2 (0.2)			
	6–8 weeks	1.0 (0.3)	1.0 (0.3)	0.0	−0.2 to 0.1	0.659
Fat mass, %	At birth	6.0 (4.4)	6.7 (6.1)			
	6–8 weeks	18.5 (5.3)	18.9 (4.5)	−0.5	−3.6 to 2.6	0.742
Fat-free mass, kg	At birth	3.4 (0.5)	3.4 (0.4)			
	6–8 weeks	4.3 (0.7)	4.2 (0.6)	−0.1	−0.4 to 0.2	0.477
Fat-free mass, %	At birth	93.8 (4.2)	93.1 (5.9)			
	6–8 weeks	81.5 (5.4)	81.1 (4.5)	0.5	−2.5 to 3.6	0.729
Muscle mass, kg	At birth	1.1 (0.2)	1.2 (0.2)			
	6–8 weeks	1.5 (0.3)	1.5 (0.2)	−0.1	−0.2 to 0.1	0.198
Hydration (TBW), %	At birth	78.2 (5.7)	77.3 (7.4)			
	6–8 weeks	66.1 (5.6)	65.4 (4.4)	0.7	−2.7 to 4.2	0.678

Data are presented as observed means and standard deviations (SDs) of outcomes measured within 72 h of delivery and at 6–8 weeks of age. Results from linear mixed model analyses are presented as estimated mean differences (estimates) in intervention group compared with control group, with corresponding 95% confidence intervals (CIs) and *p*-values.

### Appendix A.4

**Table A4:** Birth-related outcomes—Per protocol analysis.

Outcome	Control *(n* = 53)	Intervention (*n* = 23)	Relative Risk	95% CI	*p*
Normal vaginal delivery, *n* (%)	47 (89)	18 (78)	0.9	0.7 to 1.1	0.292 ^b^
Induced labor, *n* (%)	10 (19)	6 (26)	1.6	0.7 to 3.7	0.346 ^a^
Instrumental delivery, *n* (%)	2 (4)	2 (9)	2.3	0.3 to 15.4	0.580 ^a^
Caesarean section, *n* (%)	4 (8)	3 (13)	1.7	0.4 to 7.1	0.668 ^a^
Perineal tear *, grade 3–4, *n* (%)	1 (2)	1 (5)	-	-	-
Episiotomy *, *n* (%)	5 (10)	0 (0)	-	-	-
Shoulder dystocia, *n* (%)	1 (2)	0 (0)	-	-	-
Postpartum hemorrhage, *n* (%)	8 (15)	2 (9)	0.6	0.1 to 2.6	0.714 ^a^
Length of hospital stay, days	3 (2, 4)	3 (2, 4)	-	-	0.358 ^c^

Data are presented as frequencies (*n*) and percentages (%) or means and standard deviations (SDs). Results are presented as relative risks (RRs), with corresponding 95% confidence intervals (CIs) and *p*-values. ^a^ Fisher’s exact test, ^b^ Χ^2^ test, ^c^ Mann–Whitney U-test. * Only for vaginal deliveries (control, *n* = 49, intervention, *n* = 20).
