# Supplementary material for: Neonatal Outcomes Following a Preconception Lifestyle Intervention in People at Risk of Gestational Diabetes: Secondary Findings from the BEFORE THE BEGINNING Randomized Controlled Trial
Source: Nutrients. 2025 Nov 6;17(21):3492. doi: 10.3390/nu17213492 (PMC12608746; doi:10.3390/nu17213492)
Supplement: Supplementary file 1 [file nutrients-17-03492-s001.zip › nutrients-3946317-supplementary.pdf]

Supplementary File S1: Informed written consent

INVITATION TO PARTICIPATE IN A RESEARCH PROJECT

INTERVAL TRAINING AND TIME RESTRICTED EATING BEFORE AND DURING PREGNANCY

You are invited to participate in a research project where we will investigate whether the combination of high intensity exercise and time-restricted eating before and during pregnancy can reduce the risk of high blood sugar levels during pregnancy. The study will also examine whether new ultrasound technology can provide better imaging of the fetus during pregnancy. Participants have to be between 18 and 39 years old, planning a pregnancy within the next six months and meet at least one of the criteria for increased risk of gestational diabetes: 1) Body mass index (BMI) between 25 and 40 kg/m^2, 2) previous gestational diabetes, 3) a close relative with diabetes (either parents, siblings or children with diabetes), 4) ethnic origin from Asia or Africa, and/or 5) previously given birth to a child of more than 4.5 kg. You must understand written and oral English or Norwegian. The project is undertaken at NTNU, in collaboration with St. Olavs hospital.

WHAT IS THE PROJECT ABOUT?

In the project, we will measure your blood glucose (sugar) levels, insulin and lipids in the blood, body composition, physical fitness, and blood pressure at the beginning of the project and again after eight weeks. During pregnancy, we will again measure your blood glucose levels, insulin and lipids in the blood, as well as blood sugar regulation, and body composition, when you are 12 and 28 weeks pregnant.

In addition to the regular ultrasound screening in week 12 and 20 you will be invited to an ultrasound scan of the fetus in week 32. At these examinations we will also save ultrasound raw data after the measurements. The images, measurements and raw data will be used for research to test new methods for improving image quality in ultrasound imaging.

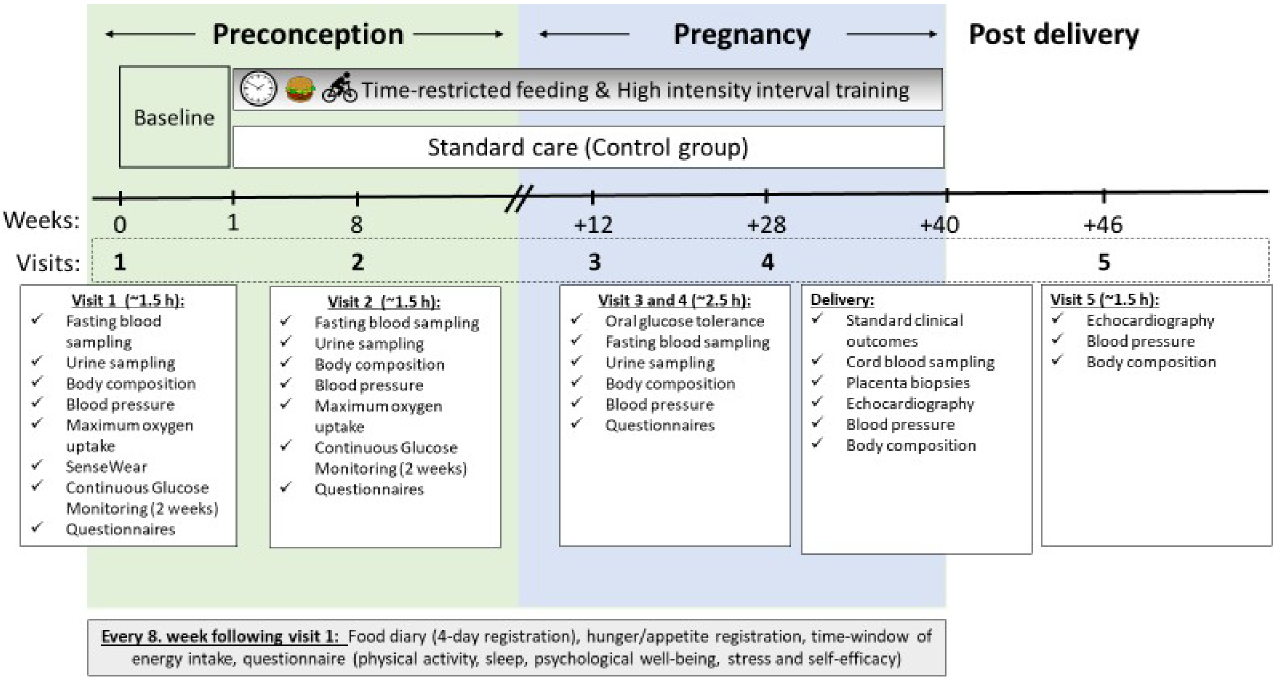

At birth, umbilical cord blood and a small piece of the placenta will be collected. You will also be asked to complete some questionnaires (regarding physical activity, sleep, and quality of life). In addition, we will obtain relevant information from

the child's birth record from the hospital. Within 3 days after birth and 6 weeks after birth, we will examine the newborn's body composition using bioimpedance. This measurement involves that we put electrodes on the baby's hands and feet and the measurements will last no longer than 5 minutes. At the same time, we will also measure the baby`s blood pressure and heart function. The two assessments before pregnancy last approximately 1,5 hours, whereas the two assessments during pregnancy last approximately 2,5 hours. The two assessments after birth last approximately 1,5 hours each.

Participants will be randomly allocated to one of two groups. One of the groups shall reduce their daily time- window of energy intake to a maximum of 10 hours per day for a minimum of five days a week. That means that you eat all meals within 10 hours, with a start-up no later than 9 am (last meal at 7 pm). In addition, this group shall exercise both before and during pregnancy. The training will be based on your level of physical fitness, and we will provide a "menu" of exercise options, as well as a more structured training program for those who want that. The training will be regulated using Personal Activity Intelligence (PAI), and the participants in this group will receive a smart watch that records PAI throughout the study period. This program will continue until pregnancy (for up to twelve months). If you become pregnant within twelve months, we will make some slightly adjusted recommendations on how to exercise during pregnancy. If you do not become pregnant within twelve months, participation in the study will be terminated. Participants who are concerned about the safety of their fetus during training, can exercise at the hospital with monitoring of the foetal heart rate during training. The second group is a control group that continues to live as usual during the study period. Participants in the control group who do not become pregnant after twelve months will be offered a similar training program as the participants in the intervention group. The project will collect and record personal information about you. This includes age, weight, height, body composition (the amount of muscle mass and body fat), your physical activity level (questionnaire and physical activity monitors), sleep and psychological well-being (questionnaire), blood pressure, results from urine – and blood analyses and continuous measurement of blood glucose. In addition, we will collect and record information about the infant. This includes information from the medical record about the baby`s weight, height and results from routine paediatric examination, umbilical cord blood, the baby's body composition, blood pressure and heart function.

You will be asked to wear a small glucose monitor for 14 days, twice during the study. The first time is at the beginning of the study period and the second is after 6-8 weeks. The monitor will be attached to your upper arm with a small, thin needle. The device will automatically measure your blood glucose levels and store these data for later analyses. You can attach this yourself or come to us at the hospital. You will also be asked to wear an activity monitor on your upper arm for 14 days at the start of the study. Every 8 weeks during the study period, you will be asked to record everything you eat and drink in an online food diary (Calorie Counter by - FatSecretApp) for 4 days (3 weekdays and 1 weekend day), answer a form about how hungry you feel and record the daily time-window of energy intake. We will also ask you to fill out a questionnaire with questions about physical activity, sleep, psychological well-being, stress and self-efficacy every 8 weeks. We will measure your blood pressure twice before you become pregnant and again twice during pregnancy. At the same time, we will also sample blood from you. From these blood samples, we will analyze cholesterol (blood lipids) and glucose (blood sugar). We will also freeze some of the blood for later analyses (analyses of insulin and other markers for metabolism). We ask for permission to store some of the blood for later analyses that are not yet determined but are likely to include markers for metabolism and inflammation which will be relevant for answering our research questions. In the umbilical cord blood, we intend to measure different markers for metabolism and heart function. We would also

like to ask for permission to store some of this blood for later analyses that are not yet determined but will be those relevant to answer our research questions in the project. You will have to come in fasted on the testing days, meaning that you have not eaten or drunk anything but water since 10 pm the night before. After initial blood sampling, you will get to drink a solution of glucose (sugar) dissolved in water and we will sample blood every 30 min for 2 hours for analyses of how your body responds to this. At each visit you will also receive a questionnaire, which you complete and return to us at the visit.

FORESEEABLE BENEFITS AND PREDICTABLE RISKS AND BURDENS OF TAKING PART

The benefits of taking part in this project are that you will get information about your blood sugar, body composition, blood pressure and physical fitness. If you become pregnant you will receive additional follow-up during pregnancy (extra ultrasound of the fetus with focus on fetal growth and the fetal heart) and cardiac examination of the new-born after birth. These additional ultrasound scans do not possess any risks or burdens except for extra the time it will take to complete them. If you are allocated to the intervention group, it can also benefit your overall health due to guided training and eating time-restricted for a limited period. The project implies that you must find time for the assessments and registrations. We see no major burdens of taking part in the project aside from potential discomfort you might feel from the blood sampling and insertion of the blood glucose sensor.

VOLUNTARY PARTICIPATION AND THE POSSIBILITY TO WITHDRAW CONSENT

Participation in the project is voluntary. If you wish to take part, you will need to sign the declaration of consent on the last page. You can, at any given time and without giving us a reason, withdraw your consent. This will not have any consequences for any future treatment. If you decide to withdraw participation in the project, you can demand that your tests and personal data concerning health is deleted, unless however, the personal data concerning health and tests have already been analysed or used in scientific publications. If you at a later point, wish to withdraw consent or have questions regarding the project, you can contact Trine Moholdt by phone: + 47 970 98 594 or by e-mail: trine.moholdt@ntnu.no.

WHAT WILL HAPPEN TO YOUR PERSONAL DATA CONCERNING HEALTH?

Any personal data concerning health that has been recorded about you will only be used as described in the purpose of the project. You have the right to access information that has been recorded about you and the right to stipulate that any error(s) in the information that is recorded is/are corrected. You also have the right to know which security measures have been/will be taken when your personal data concerning health is processed.

All information will be processed and used without your name or personal identification number, or any other information that is directly identifiable to you. A code links you and your personal data concerning health via an identifier list. Only project manager Trine Moholdt and project assistant Guro Rosvold will have access to this list.

In order to investigate the long-term impact of the study intervention, we ask for permission to keep the data for 20 years and to contact the participants again for any follow-up studies on you and your child's health in the years after birth. If we wish to use the biological material and health information from the child within 20 years after data collection, we will obtain a signed consent from the child when it is competent to sign such a consent (16 years old).

SHARING OF PERSONAL DATA AND TRANSFER OF PERSONAL DATA ABROAD

By agreeing to participate in the study, you are also consenting to that your information can be transferred to another country as a part of research collaboration and publication. This can be a country where the laws do not meet the requirements of the European Data Protection Law. The project manager will therefore ensure that your personal data concerning health is kept safe.

At present, we have only planned to share the ultrasound data with our partner GE HealthCare abroad (see more details below). We have not planned to share other data from the study abroad, but this may become relevant at a later point in the project period.

The code that connects you and your personal data concerning health will not be released.

NTNU and St. Olav's hospital, in collaboration with GE HealthCare, are performing research on new technology for improving image quality when using ultrasound in fetal imaging. If you agree to this, the de-identified ultrasound data will be shared with NTNU and GE HealthCare for future research and as part of further developing technology of commercialization within GE HealthCare

WHAT WILL HAPPEN TO THE TESTS YOU HAVE TAKEN?

The blood and urine samples taken from you, as well as the placenta biopsies and umbilical cord blood from the infant, will be stored in a specific Research Biobank connected to the Research Project. Responsible for this is project manager Trine Moholdt. The blood samples, urine, and umbilical cord blood will be physically stored at Department of Circulation and Medical Imaging, NTNU.

The Research Biobank will be terminated once the research project has ended.

It may be of interest to send samples abroad for analyses in relation to this project. Currently we are not sure if this will happen, or in which country the samples will be analysed. If sample material is sent abroad, the residual material after analysis will either be returned to the biobank in Trondheim or destroyed at the end of the project.

INSURANCE

Participants are covered by the Patient Injuries Act.

FOLLOW-UP PROJECT

A follow-up project may be of interest. We therefore ask for permission to retain your contact information in case we wish to contact you again later. You can join the project without giving us permission to contact you later.

FINANCE

The expenses of the project will be covered from The European Foundation for the Study of Diabetes/NovoNordisk Foundation and The Liaison Committee for Education, Research and Innovation in Central Norway. The project is also financed by the Centre of Innovative Ultrasound Solutions (CIUS), a large research and innovation project lead by NTNU: CIUS has over 20 collaboration partners where the research council of Norway, Helse Midt-Norge, St Olavs Hospital, NTNU and GE HealthCare are some of the contributors. One of the researchers in the project (Svein Erik Måsøy) has a 20% position at GE HealthCare, this has been clarified and approved by NTNU. Otherwise, none of the researchers in this project have any financial ties to GE HealthCare.

Participation in the project is free of charge.

APPROVAL

The Regional Committee for Medical and Health Research Ethics has reviewed and approved the Research Project (REK 2020/143756).

In accordance with the General Data Protection Regulation the controller NTNU and the project manager Trine Moholdt is independently responsible to ensure that the processing of your personal data concerning health has a legal basis. This project has legal basis in accordance with the EUs General Data Protection Regulation, article 6 no. 1a, article 9 no. 2a and your consent.

You have the right to submit a complaint on the processing of your personal health data concerning health to the Norwegian Data Inspectorate.

CONTACT INFORMATION

If you have any questions regarding the research project, you can contact Trine Moholdt, + 47 970 98 594, trine.moholdt@ntnu.no.

You can contact the Institution's Data Protection Officer if you have any questions related to the use of your personal health data concerning your health in the research project. The officer to contact is Thomas Helgesen,
+ 47 930 79 038, personvernombud@ntnu.no.

I CONSENT TO PARTICIPATING IN THE RESEARCH PROJECT AND THAT MY PERSONAL DATA CONCERNING HEALTH AND BIOLOGICAL MATERIAL CAN BE USED AS DESCRIBED ABOVE

Please tick relevant boxes below:

*I **approve** that my contact information may be retained in case the researchers contact me for any follow-up studies:* □

*I do **not approve** that my contact information may be retained in case the researchers contact me for any follow-up studies:* □

*I **approve** that de-identified ultrasound data may be shared with GE HealthCare:* □

City/Town and Date — Participant's Signature

Participant's Name (in BLOCK LETTERS)

Supplementary File S2: Exercise options

Before The Beginning

Exercise Options

EXERCISE IN PREGNANCY

Good cardiorespiratory fitness and strong muscles, joints and skeleton provide better conditions for a healthy pregnancy. During pregnancy, your workout should contain the same elements as before the pregnancy, but with a slightly lower intensity level.

Avoid any activities that has a lot of jerky, bouncing movements that may cause you to fall, like horseback riding, downhill skiing, off-road cycling, or gymnastics. Also, avoid sports in which you may be hit in the belly, such as ice hockey, boxing, soccer, or basketball.

Below you will find suggestions for workouts and exercise programs both before - and during pregnancy. These are only suggestions, and you decide for yourself what kind of physical activity you want to do. We are different and have different preferences for which activities are pleasurable and gives you joy.

WHAT IS TRAINING INTENSITY?

Training intensity is the amount of effort you are putting into whatever exercise you are doing. You can subjectively rate your level of exertion during exercise by using Borg scale. The figure below will help you evaluate where on the scale you are during exercise. Vigorous-intensity exercise is a physical activity done with a large amount of effort, resulting in a substantially higher heart rate and rapid breathing. Engaging in vigorous physical activity can provide many health advantages. Thus, it can be useful to know the distribution between light -, moderate – and vigorous intensity during your workouts. Evaluate the intensity while doing the activity. Try to be as sincere as possible. Remember that this is a subjective scale – what others thinks is irrelevant. Use the full scale and choose the number that suits your experience during activities best.

Borg`s Rating of Perceives Exertion (RPE) Scale

Value	Perceived Exertion Rating	Description of Exertion
6	No exertion	Rest
7	Extremely light	
8		
9	Very light	Comfortable walking pace. Conversation is easy.
10		
11	Light	
12		
13	Somewhat hard	You feel you could run/walk for a while at this pace. Still able to talk.
14		
15	Hard	Hard, but you`re not struggling. You can talk but not in full sentences.
16		
17	Very hard	Very hard – Starting to get uncomfortable and you`re getting tired.
18		
19	Extremely hard	Extremely hard. Your body is screaming at you to stop. You can no longer talk because your breathing is heavy.
20	Maximal exertion	Max exertion.

7-11 = Light intensity **12-15 = Moderate** intensity **16-20 = Vigorous** intensity

PAI

To measure your activity level, we use the science-backed health score called PAI (Personal Activity Intelligence). You earn PAI points every time your heart rate increases: The higher your heart rate, the faster you earn PAI. Research shows that those who achieve 100 PAI or more every week over time live on average eight years longer than others.

PAI considers your age, your gender, your resting heart rate and your maximum heart rate. In other words, PAI is not based on the number of steps you walk or how many minutes of physical activity you perform each day. Those measurements do not consider the intensity of the activity. Therefore, we think that PAI is a more accurate and attractive activity standard to tell you if you actually exercise enough.

Your goal is to earn 100 PAI or more during a 7-day period. It doesn't matter what type of activity you do to earn PAI – you could walk, run, cycle, row, swim or go skiing. All that matters is how high your heart rate is during the activity.

PAI is based on the only thing that reflects the intensity of your activity: your heart rate. Everything you need to do to use PAI is to measure your heart rate continuously. The better your fitness is, the more physical activity is needed to achieve 100 PAI. In other words, PAI adjusts to your progress. If you are untrained and out of shape, you could earn your 100 PAI just by going for short walks regularly throughout the week, as that will raise your heart rate. If you are in shape and well trained, you will need to do more.

It is easier to reach the first 50 PAI compared to the next 50. That is because the risk reduction for lifestyle diseases is greatest when progressing from total inactivity to some physical activity. This means that if you repeat a workout two days in a row, you will get less PAI on the second workout.

Remember to turn on the **"workout-mode"** during exercise.

ENDURANCE: BEFORE PREGNANCY

Endurance sessions can be done both outside and indoors. The sessions can be done with any endurance equipment: Bicycle, treadmill, skiers, rowing machine, assault bike or elliptical machine.

HIGH-INTENSITY INTERVAL TRAINING 1	
**What?**	**Duration**
**Warm-up: 5 minutes at low intensity (Borg scale: 7-11), thereafter 5 minutes with moderate intensity (Borg scale 12-15).**<br><br>**Interval: Four 4-min work-bouts at high intensity (Borg scale 16-20). 3 minutes active break after each work-bout at low-to-moderate intensity.**<br><br>**Cool-down: 5 minutes with low intensity (Borg scale: 7-11).**	40 min

HIGH-INTENSITY INTERVAL TRAINING 2	
**What?**	**Duration**
**Warm-up: 5 minutes at low intensity (Borg scale: 7-11), thereafter 5 minutes with moderate intensity (Borg scale 12-15).**<br><br>**Interval: Ten 1-min work-bouts at high intensity (Borg scale: 16-20). 1-minute low-intensity break after each work-bout.**<br><br>**Cool-down: 5 minutes at low intensity (Borg scale: 7-11).**	35 min

TABATA: BEFORE PREGNANCY

Tabata is a type of high-intensity interval training (HIIT) workout in which you can combine high-intensity endurance training with strength training. Tabata is a 4-min period consisting of 8 rounds of 20 seconds of work at maximum effort, followed by 10 seconds of rest. Between these 4-min periods, you have 30 sec of rest. Target intensity is high (Borg scale: 16-20). Below, you find one example of a Tabata session.

TABATA			
**Equipment: Kettlebells, yoga mat, bench/step/box, weight plates or dumbbells**			
**Exercises**	**Muscle group**	**Comment**	**Duration**
**Station 1: Walking burpees**	Thighs and gluteal muscle	Begin standing with your feet hip-distance apart (A). Then, squat down and step one foot back at a time (B). After you're in the plank position (C), walk your feet back to meet your hands and return to standing. That's one repetition	34 min
**Station 2: Reverse crunches**	Abdominal muscles	Start lying down with your arms by your sides. Raise your legs so your thighs are perpendicular to the floor and your knees are bent at a 90° angle. Breathe out and contract your abs to bring your knees up towards your chest and raise your hips off the floor. Hold for a beat in this position, then slowly lower your legs back to the starting position.	
**Station 3: Bent over row w/kettlebells**	Back and shoulders	With a kettlebell (or another type of weight) in each hand, bend over at about a 45-degree angle. Keep the back straight throughout the exercise. Contract your abdominal muscles and breathe in. Lift the weights straight up, exhaling. While lifting, the arms should go no higher than parallel with the shoulders—slightly lower than the shoulders is fine. While lifting, try to keep the wrists from excessive extra movement down or to the side. The legs should be kept still throughout the exercise. Lower the weights in a controlled manner while inhaling. Remain bent over until all repetitions are complete.	
**Station 4: Push-ups**<br><br>**Modifications:**<br>• **Push-ups against a wall or bench, or on your knees**	Chest and arms	Get down on all fours, placing your hands slightly wider than your shoulders. Straighten your arms and legs.<br>Lower your body until your chest nearly touches the floor/wall/bench. Pause, then push yourself back up.<br>Repeat.	
**Station 5: Farmers walk w/kettlebells**	Thighs, gluteal muscle, calves, back, neck, arms	Pick up a pair of kettlebells (or another type of weight) in each hand. Stand tall with kettlebells out to the sides a couple of inches (do not let the weight touch your legs)<br>Look straight ahead and pinch your shoulder blades (scapula) back slightly. Keep your core tight. Walk slowing and with short, deep steps.	
**Station 6: Biceps curl and shoulder press w/dumbbells**	Arms and shoulders	Holding a pair of dumbbells (or another type of weight), stand tall with your feet shoulder-width apart. Make sure your core is tight, and your chest is up. Begin by curling the weight up towards your shoulders. Keep your upper arms tight at your sides.<br>Once the dumbbells reach your shoulders, twist the dumbbells to have your palms face out. Now, push the dumbbells overhead. Slowly, lower the dumbbells to your shoulders, then flip them back so your palms are facing you. With arms tight at your sides, lower the dumbbells to the starting position.	
**Station 7: Box step-ups**	Thighs and gluteal muscle	Place your right foot on the box, lean forward and step up so you're standing with both feet on the box. Then, step back with your right foot and place it on the ground. Then, step back with	

		your left foot. Next, step up with your left foot followed by the right foot. Switch foot (right/left) with each repetition.	
**Stations 8: Russian twist**	Stomach muscles	Sit on your sit bones as you lift your feet from the floor, keeping your knees bent. Elongate and straighten your spine at a 45-degree angle from the floor, creating a V shape with your torso and thighs.<br>Reach your arms straight out in front, interlacing your fingers or clasping your hands together. Use your abdominals to twist to the right, then back to center, and then to the left. This is 1 repetition. If you want, you can hold a dumbbell (or another type of weight) in your hands.	

Warm-up: 10 minutes with low intensity (Borg scale: 7-11) on endurance equipment of choice.

Cool-down: 5 minutes with low intensity (Borg scale: 7-11) on endurance equipment of choice.

EXERCISE PROGRAM: BEFORE PREGNANCY

What your weekly training plan could look like:

EXERCISE PROGRAM			
**Day**	**What?**	**Intensity (Borg scale)**	**Duration**
**Monday**	**Warm-up:** 5 minutes with at intensity, thereafter 5 minutes with moderate intensity.<br>**Intervals:** 4 x 4 min high-intensity interval training, with 3 min rest between the 4-min work-bouts. It is smart to use uphill walking/running. If outside: The break will be walking down to the bottom of the hill.<br>**Cool-down**: 5 min with low intensity.	During the 4-min work-bouts, you should be between 16-20 on the Borg scale.	Ca. 40 min
**Tuesday**	Rest		
**Wednesday**	**Warm-up:** 5-10 minutes increasing your pace and heart rate gradually.<br>**Workout:** 50 minutes distance training with a moderate pace/intensity.<br>**Cool-down**: 5 min with low intensity.	12-15 on Borg scale.	Ca. 60 min
**Thursday**	Rest		
**Friday**	**Warm-up:** 5 minutes with at intensity, thereafter 5 minutes with moderate intensity.<br>**Intervals:** 4 x 4 min high-intensity interval training, with 3 min rest between the 4-min work-bouts. It is smart to use uphill walking/running. If outside: The break will be walking down to the bottom of the hill.<br>**Cool-down**: 5 min with low intensity.	During the 4-min work-bouts, you should be between 16-20 on the Borg scale.	Ca. 40 min
**Saturday**	Rest		
**Sunday**	Rest		

ENDURANCE: DURING PREGNANCY

Endurance sessions can be done both outside and indoors. The sessions can be done with any endurance equipment: Bicycle, treadmill, skiers, rowing machine, assault bike or elliptical machine.

WORKOUT 1	
**What?**	**Duration**
**Warm-up: 5 minutes with low intensity (Borg scale: 7-11)**<br><br>**Workout: 50 min with moderate intensity (Borg scale: 12-15)**<br><br>**Cool-down: 2-3 minutes with low intensity (Borg scale: 7-11)**	Ca. 60 min

WORKOUT 2	
**What?**	**Duration**
**Warm-up: 5 minutes with low intensity (Borg scale: 7-11), thereafter 5 min med moderate intensity (Borg scale 12-15).**<br><br>**Workout: Every second minute do a 30 second sprint with high intensity (Borg scale 16-17). Repeat until you have completed 5-10 of these sprints.**<br><br>**Cool-down: 5 minutes with low intensity (Borg scale: 7-11**	Ca. 25-35 min

TABATA: DURING PREGNANCY

Tabata is a type of high-intensity interval training (HIIT) workout in which you can combine high-intensity endurance training with strength training. Tabata is a 4-min period consisting of 8 rounds of 20 seconds of work at maximum effort, followed by 10 seconds of rest. Between these 4-min periods, you have 30 sec of rest. Target intensity moderate (Borg scale: 12-15). Below, you find one example of a Tabata session.
The heart rate should be no higher than 85 % of your maximum heart rate.

TABATA			
**Equipment: Dumbbells with different load and a yoga mat**			
**Exercise**	**Muscle group**	**Comment**	**Duration**
**Station 1: Air squats**<br><br>**Modifications:**<br>• **Wall ball squat**<br>• **Squat to bench/box**	Thighs and gluteal muscle.	Stand with feet shoulder-width apart, toes pointed slightly outward. Engage core muscles and pull shoulder blades together slightly to push out your chest.<br>Squat back as if you were about to sit in a chair. Keep your weight in your heels so you don’t lean forward. Your hips should move down and back.<br>Make sure your lower back curve is maintained and keep your heels flat on the floor the whole time. Hips will descend lower than knees. (The eventual goal is to touch your glutes to the backs of your calves.) Hold for a few seconds, then rise up by pushing through heels and using glutes to return to a standing position.	34 min
**Station 2: Glute Bridge**	Thighs, gluteal muscle, abdominal	Start flat on your back with your legs bent at a 90-degree angle and feet placed flat on the ground. Make sure your toes are turned outward at 45-degree angles and your knees are facing in the same direction as your	

**Modification: skip this exercise if you are uncomfortable when lying on your back.**	muscles and back.	toes. Drive down through your feet and push your hips up. You should feel this variation fatiguing the outer portion of your thighs. Make sure you keep your knees over your toes throughout the entire movement. Don’t let them move forward over the toes. In a controlled motion, let your hips sink back down toward the ground. This completes 1 repetition.	
**Station 3: Side plank**<br><br>**Modifications:**<br>• **Sideplank with bent knees**	Abdominal muscles and back.	Lie on your right side, legs extended and stacked from hip to feet. The elbow of your right arm is directly under your shoulder. Ensure your head is directly in line with your spine. Your left arm can be aligned along the left side of your body.<br>Engage your abdominal muscles, drawing your navel toward your spine. Lift your hips and knees from the mat while exhaling. Your torso is straight in line with no sagging or bending. Hold the position. 4 times on each side.	
**Station 4: Diagonal lift, standing on all fours**	Back, gluteal muscle and thighs.	Stand on all fours with your head extending your body. Flex your abdomen and lower back to stabilise. Alternately stretch one arm and the opposite leg to form extensions of your body.	
**Station 5: Push-ups**<br><br>**Modifications:**<br>• **Push-ups against wall/bench**<br>• **Push-ups on your knees**	Chest and arms	Get down on all fours, placing your hands slightly wider than your shoulders. Straighten your arms and legs.<br>Lower your body until your chest nearly touches the floor/wall/bench. Pause, then push yourself back up.<br>Repeat.	
**Station 6: Standing alternating dumbbell curls**<br><br>**Modifications:**<br>• **Sitting dumbbell curls**	Arms.	Stand with your feet shoulder-width apart and holding a dumbbell in each hand with an overhand grip, with your palms facing your sides. This is your starting position. Raise one dumbbell toward your shoulder while simultaneously rotating the back of your hand. Pause with your palm facing your shoulder. Reverse the movement to lower the weight to the starting position.	
**Station 7: Dumbbell bent over row on bench**	Back.	Choose a flat bench and place a dumbbell on each side of it. Place the right leg on top of the end of the bench, bend your torso forward from the waist until your upper body is parallel to the floor, and place your right hand on the other end of the bench for support. Use the left hand to pick up the dumbbell on the floor and hold the weight while keeping your lower back straight. The palm of the hand should be facing your torso. This will be your starting position. Pull the resistance straight up to the side of your chest, keeping your upper arm close to your side and keeping the torso stationary. Breathe out as you perform this step. Lower the resistance straight down to the starting position. Breathe in as you perform this step. Repeat the movement for the specified number of repetitions. 4 times on each arm.	

**Station 8: Dumbbell lateral raise**<br><br>**Modifications**<br>• **Sitting.**	Shoulders.	Stand or sit with a dumbbell in each hand at your sides. Keep your back straight, brace your core, and then slowly lift the weights out to the side until your arms are parallel with the floor, with the elbow slightly bent. Then lower them back down, again in measured fashion – you'll find it all the harder if you avoid speeding up. A lot of people will cheat by "shrugging" the weights up using their traps. Resist the urge to do that by not raising your shoulder blades during the rep – instead focus on the delts.	

EXERCISE PROGRAM: DURING PREGNANCY

What your weekly training plan could look like:

**EXERCISE PROGRAM**			
**Day**	**What?**	**Intensity (Borg scale)**	**Time**
**Monday**	**Warm-up:** 5-10 minutes increasing your pace and heart rate gradually.<br>**Workout:** 50 minutes over distance training with a moderate pace.<br>**Cool-down**: 5 min with low intensity.	12-15 on Borg scale.	Ca. 60 min
**Tuesday**	Rest		
**Wednesday**	**Warm-up:** 5 minutes with low intensity (Borg scale: 7-11), thereafter 5 min med moderate intensity (Borg scale 12-15).<br>**Workout:** Every second minute do a 30 second sprint with high intensity (Borg scale 16-17). Repeat until you have completed 5-10 intervals.<br>**Cool-down:** 5 minutes with low intensity (Borg scale: 7-11	16-17 on Borg scale.	Ca. 25-35 min
**Thursday**	Rest		
**Friday**	**Warm-up:** 5-10 minutes increasing your pace and heart rate gradually.<br>**Workout:** 50 minutes over distance training with a moderate pace.<br>**Cool-down**: 5 min with low intensity.	12-15 on Borg scale.	Ca. 60 min
**Saturday**	Fri		
**Sunday**	Fri		
